# 

**Published:** 1936

**Authors:** 


A. RENDLE SHORT, M.D., B.S., B.Sc., F.R.C.S.

E. WATSON-WILLIAMS, M.C., Ch.M., F.R.C.S., *Assistant Editor.*

*Editorial Secretary:* A. L. FLEMMING, M.B., Ch.B.

### CONTENTS.

| | Page |
|---|---|
| Corneal Transplantation: Its History, Development and Practice. By J. W. Tudor Thomas, D.Sc., M.D., M.S., F.R.C.S. | 75 |
| A Treatment for Sciatica. By A. Rendle Short, M.D., B.S., B.Sc., F.R.C.S. .. .. .. .. .. .. .. .. | 87 |
| Reviews of Books .. .. .. .. .. .. .. .. | 101 |
| Editorial Note .. .. .. .. .. .. .. .. | 117 |
| Meetings of Societies .. .. .. .. .. .. .. | 118 |
| The Medical Library of the University of Bristol .. .. | 119 |
| Publications Received .. .. .. .. .. .. .. | 121 |
| Local Medical Notes .. .. .. .. .. .. .. | 122 |

